# Reclaiming Power and Partnerships in the Wake of Donor Retraction *through South–South Partnership*

**DOI:** 10.1111/disa.70009

**Published:** 2025-08-29

**Authors:** Basma Taysir El Doukhi

**Affiliations:** ^1^ University of Kent; Community Resilience at British Red Cross

**Keywords:** decolonisation of aid, donor retraction, funding models, geopolitical instability, Global South, humanitarian aid, locally driven development, resilience, South–South partnership, sustainable development

## Abstract

The recent US foreign assistance cuts represent not only a funding crisis but also a reckoning for the humanitarian and development sectors. This moment must catalyse a shift towards South–South cooperation, partnership and funding ecosystems that are not beholden to the strategic fluctuations of traditional donors. Global North institutions continue to marginalise the knowledge, leadership and capacities of Global South communities; thus, aid needs to be reshaped as solidarity, not charity. This paradigm shift would place communities, civil society and local governments at the centre of decision‐making, reclaiming power, dignity and autonomy by local and displaced actors. To make this transition effective, national and local communities and actors must invest in capacity strengthening, inclusive governance models and equitable partnerships. Embracing these practices will not only increase the sustainability of interventions but also uphold the dignity and leadership of those at the frontlines of crisis and humanitarian action. In addition, it is imperative to reform existing aid architectures to reflect new geopolitical and environmental realities.

The recent US foreign assistance cuts represent not just a funding crisis, but also a reckoning for the humanitarian and development sectors. The loss of funding for 79 million people exposes the fragility of a system still deeply dependent on the priorities of powerful donors. The cuts have also disproportionately affected the very actors the localisation agenda promised to empower: national NGOs, displaced led organisations and community‐based actors. According to an OCHA survey (2025), 80% of RLOs and women‐led organisations have had their project funding terminated; 41% of respondents reported funding cuts that affected their ability to sponsor, support and advance their partnerships and collaboration agreements with national and local actors.

At a moment when global crises are becoming increasingly complex – intertwining conflict, climate, displacement and economic collapse – we cannot afford to retreat from local knowledge and leadership. Instead, we must radically reimagine humanitarianism: not as a pipeline of aid from North to South, but as a collaborative infrastructure rooted in trust, accountability and shared vision.

Building on the UN Secretary‐General's report for the World Humanitarian Summit (WHS) in 2016, the humanitarian sector must ‘reinforce, not replace’ local systems. The need to support and capitalise on local, national and regional actors in humanitarian action was highlighted throughout the WHS consultation process and clearly captured in the Secretary‐General's report: recommendations outlined in the report to ‘commit to as local as possible and as international as necessary’ and to ‘put people at the center’ reaffirmed local ownership and leadership, including representation.

As a researcher and humanitarian practitioner shaped by the lived experience of displacement, I argue that this moment must catalyse a shift towards South–South cooperation, partnership and funding ecosystems that are not beholden to the strategic fluctuations of traditional donors. We must invest in relationships with non‐traditional actors – regional philanthropies, local civil society networks and community‐based coalitions – that are committed to people‐led systems of care and support. This also demands that donors listen differently: not to audit and extract, but to accompany and affirm.

I see the need for a South–South movement and a shift towards more accountable, locally driven priorities: ones that speak the language and reflect the needs of the communities we serve, work with and belong to. This movement challenges traditional donor–recipient models and opens pathways for equitable partnerships and knowledge exchange rooted in shared lived realities. This brings both unique opportunities and critical challenges that demand reflection, humility and sustained commitment.

## 1 SOUTH–SOUTH PARTNERSHIP AND LOCALLY DRIVEN AGENDAS

Some of the most powerful opportunities for South–South partnership lie in enabling Southern actors to own and guide humanitarian and development agendas. This is a crucial shift that introduces more relevant, context‐specific and sustainable solutions. When development and humanitarian priorities are locally owned, they build autonomy and strengthen the capacity of national and local systems. This, in turn, encourages mutual dependence among Southern actors on their own expertise and leadership, advancing self‐determination and resilience.

South–South partnership fosters trust and transparency about what can and cannot be delivered, resetting relationships towards more equitable terms. It also opens the door for new and diverse sources of funding – from private sector actors, regional donors and diaspora communities – who are invested in achieving national development goals. In the face of global funding volatility, particularly recent US foreign assistance cuts, this shift is not only strategic but also essential to ensuring the durability and dignity of local humanitarian and development systems.

A growing body of evidence and practice across the Global South points to the need to move away from traditional, donor‐led approaches and towards partnership‐based models rooted in mutual accountability and locally defined priorities. The shift towards locally led development places communities, civil society and local governments at the centre of humanitarian and development decision‐making, transforming them from passive recipients of aid into strategic partners and rights‐holders. This reframing fosters more equitable, contextually grounded and sustainable responses to crises, particularly in complex displacement settings like Turkey and Syria.

Recognising refugee or migrant‐led organisations (R/MLOs) as core partners rather than implementers or beneficiaries aligns with this paradigm shift by centring community knowledge and generating practical tools to support more collaborative, inclusive models of urban and ecological resilience. This approach resonates with broader South–South cooperation principles and offers actionable insights for funders and practitioners seeking to operationalise truly locally driven humanitarianism.

A critical shift is under way in the humanitarian and development sectors towards the decolonisation of aid – a process that involves not only the redistribution of resources but also, and more importantly, the reclaiming of decision‐making power by local and displaced actors. This transformation challenges longstanding power hierarchies dominated by Global North institutions and confronts the epistemic violence that continues to marginalise the knowledge, leadership and capacities of Global South communities, supporting a broader movement to reshape aid as solidarity, not charity.

## 2 RISKS AND RECOMMENDATIONS FOR SOUTH–SOUTH PARTNERSHIP

While South–South cooperation presents timely opportunities to foster autonomy, relevance and contextual innovation in the humanitarian and development sectors, it also introduces complex challenges. Traditional donors may hesitate to shift funding towards agendas that diverge from their own strategic priorities. Additionally, some local actors may face limitations related to governance structures, accountability mechanisms and capacity to manage large‐scale interventions. These gaps – if unaddressed – could risk undermining project implementation and the effectiveness of support delivered to communities.

To mitigate these risks and fully harness the transformative potential of South–South cooperation, I recommend the following:

1. Invest in institutional capacity‐building for local civil society organisations, particularly in project governance, monitoring and financial accountability.
2. Promote inclusive dialogue and participatory planning to ensure development priorities reflect the voices of diverse, marginalised and displaced communities.
3. Strengthen accountability and transparency frameworks that build mutual trust between funders, partners and communities, including robust complaints and oversight mechanisms.
4. Encourage blended financing models, combining support from public, private and diaspora sources, while aligning with nationally defined development strategies.
5. Foster regional knowledge‐sharing and cooperation, enabling actors to pool resources and share best practices in navigating localised and cross‐border challenges.

Embedding these practices into the evolving architecture of South–South humanitarian and development cooperation will not only increase the sustainability of interventions but also uphold the dignity and leadership of those at the frontlines of crisis and humanitarian action.

If we are serious about the commitments made at the World Humanitarian Summit – to be ‘as local as possible, as international as necessary’ – then we must begin by trusting those who have been consistently sidelined yet continue to lead. Displaced and migrant organisations, women‐led and grassroots organisations do not need saving. They need resourcing. They do not need representation. They need recognition. They need radical courage and change in how we believe in their value and impact, demonstrated in funding as one of many ways.

This is not simply about repairing what's been broken. It is about building something fundamentally different, context‐specific – and more just – in its place. It requires political will, long‐term partnerships and a commitment to equity and mutual respect. The South–South cooperation paradigm in humanitarian and development action must be more horizontal, localised, integrated and adaptive. This shift is necessary and urgent, given the compounding crises of displacement, climate change, funding cuts and geopolitical instability. To make this transition effective, national and local communities and actors must invest in capacity strengthening, inclusive governance models and equitable partnerships. Simultaneously, it is imperative to reform existing aid architectures to reflect new geopolitical and environmental realities. It is a new call for funding systems that move from transactional support to transformational solidarity, rooted in mutual trust, shared knowledge and long‐term vision.

## Data Availability

Data sharing not applicable to this article as no datasets were generated or analysed during the current study.

## References

[disa70009-bib-0001] OCHA (2025) US funding freeze global survey, available at https://humanitarianaction.info/document/us-funding-freeze-global-survey/article/us-funding-freeze-global-survey-round-2-interactive-dashboard

[disa70009-bib-0002] UN (2016), Outcome of the World Humanitarian Summit: report of the Secretary‐General available at https://digitallibrary.un.org/record/842411?v=pdf

